# Prevalence of Depression in Patients with Type 1 Diabetes between 10 and 17 Years of Age in Jordan

**DOI:** 10.1155/2023/3542780

**Published:** 2023-02-22

**Authors:** Abeer Alassaf, Lobna Gharaibeh, Rajai O. Zurikat, Ala'a Farkouh, Sarah Ibrahim, Ayman A. Zayed, Rasha Odeh

**Affiliations:** ^1^Department of Pediatrics, The University of Jordan, Amman, Jordan; ^2^Pharmacological and Diagnostic Research Center, Faculty of Pharmacy, Al-Ahliyya Amman University, Amman, Jordan; ^3^Department of Internal Medicine, The University of Jordan, Amman, Jordan

## Abstract

**Methods:**

The study was conducted by distributing the Center for Epidemiological Studies Depression Scale for Children (CES-DC) to adolescents with T1D seen at Jordan University Hospital between February 2019 and February 2020. Demographic, clinical, and socioeconomic data were collected using electronic clinical charts. Possible predictors of depression were assessed using logistic regression analysis.

**Results:**

A total of 108 children were enrolled in the study with mean age of 13.7 ± 2.3 years. Fifty-eight children (53.7%) had a CES depression score less than 15, and 50 children (46.3%) had a depression score of 15 or more. The number of diabetes-related hospital admissions and the frequency of self-monitoring of blood glucose (SMBG) were significantly different between the two groups. In the multivariable analysis, both gender and SMBG frequency were statistically significant. Girls were more likely to have a depression score ≥ 15 (OR = 3.41, *p* = 0.025) than boys. Patients who were rarely testing blood glucose levels were more likely to have a depression score ≥ 15 compared to those who were testing regularly (OR = 36.57, *p* = 0.002).

**Conclusion:**

The prevalence of depressive symptoms is relatively high in adolescents with T1D, especially in those living in developing countries. Longer diabetes duration, higher glycated hemoglobin level, and less frequent blood glucose monitoring are associated with higher depression scores.

## 1. Introduction

Type 1 diabetes (T1D) is one of the most common chronic diseases in children and adolescents, with possible serious short- and long-term complications including diabetic ketoacidosis (DKA) and microvascular and macrovascular complications [1, 2]. Optimum glycemic control can reduce long-term complications [3]. This requires continuous and diligent diabetes care by patients and their caregivers, including administering insulin, frequent self-monitoring of blood glucose (SMBG), dietary compliance, and adjusting insulin doses according to meals and other factors [4]. Patients with T1D and their parents are required to be continuously vigilant for blood glucose levels and to react quickly in cases of hypoglycemia and hyperglycemia, which is quite stressful and demands extensive efforts with a significant impact on their psychosocial well-being [5]. Unfortunately, significant proportions of adolescents are not engaged in appropriate diabetes self-care [6], and many of them do not achieve the glycated hemoglobin (HbA1c) target levels [7].

Adolescence is a transitional period during which adolescents should cope with and manage general life stress [5]. Older children and adolescents with T1D face additional stress related to the continuous complex demanding diabetes care [8], which may result in psychological issues like depressive symptoms [9].

The inadequate efforts done in the field of psychosocial care and support for patients with T1D [10] may explain the suboptimal glycemic control in a significant number of patients, despite the significant advances in types and modes of insulin delivery and in the technologies used for blood glucose monitoring [11].

Children and adolescents with T1D are at higher risk for psychosocial disorders than their peers, who do not suffer from this chronic disease [12, 13]. Depression itself would result in less involvement in diabetes self-care and may be a cause of suboptimal glycemic control [14, 15]. The presence of depressive symptoms is common in adolescents, mainly in the late adolescence period, and it is especially more common in those with T1D [16, 17].

Regular screening for depressive symptoms in patients with T1D is extremely important as part of the routine clinic visits for early identification of depressive symptoms and subsequent referral for psychiatric evaluation and management [18].

The association between depression and T1D has been studied previously [13, 19], but relatively fewer studies were done in developing countries [20, 21], in which most of those studies showed a higher prevalence of depressive disorders compared to many studies done in developed countries, which is expected for many reasons including limited financial and health care resources.

We aim to explore the prevalence of depressive symptoms and the effect on metabolic control in adolescents with T1D in Jordan, as an example of a developing country with limited resources. In addition, we aim to study the association of depressive symptoms with demographic, clinical, and socioeconomic factors and diabetes care practices, which can help in the early identification of high-risk patients and hence apply early monitoring and intervention in order to decrease unwarranted complications.

## 2. Methods

This is a cross-sectional study to assess depressive symptoms in patients with type 1 diabetes, seen at the pediatric endocrine clinic in Jordan University Hospital, which is a major referral center, between February 2019 and February 2020. The diagnosis of type 1 diabetes was based on clinical symptoms and supported by autoantibody positivity. Patients eligible for the study were those who were diagnosed with type 1 diabetes for at least one year, aged between 10 and 17 years, and did not have other chronic illnesses or developmental delay. Our study was conducted after the approval of Jordan University Hospital institutional review board (IRB), according to the Declaration of Helsinki. Assessment of depressive symptoms in our cohort was through the Center for Epidemiological Studies Depression Scale for Children (CES-DC), which is a modified version of the Center for Epidemiologic Studies Scale to be used in older children and adolescents [22, 23]. It is one of the commonly used self-report depression questionnaires which consists of 20 questions to rate depressive symptoms over the previous week. Each question scores from 0 to 3. The total score ranges from 0 to 60. The total scores were grouped into less than 15 and 15 or more; the latter is suggestive of significant depressive symptoms [22]. CES-DC was distributed to patients with diabetes at our clinic, after taking their assent and their parents' consent. It was filled out by the patient, and help was offered if clarification of any item in the questionnaire was needed by the research assistant or the pediatric endocrinologist in the clinic.

Demographic and clinical data were collected from the electronic clinical charts and included age, gender, duration of diabetes, type of insulin regimen, which included multiple (long and rapid-acting insulin) or triple dose injection (intermediate and short-acting insulin), and the number of diabetes-related hospital admissions including diabetic ketoacidosis and hyperglycemia. The hospital admission rate was calculated by dividing the number of diabetes-related hospital admissions by the duration of diabetes to have a more precise idea of the frequency of admissions related to the duration of diabetes. Age-standardized body mass index (BMI) was calculated and expressed as a BMI standard deviation score (SDS). Socioeconomic data included parental age; educational level which was divided into elementary school, high school, and higher education (diploma, bachelor's degree, master's degree, and PhD); and marital status. Additional data included the number of siblings, type of insurance, and family monthly income classified into low-, middle-, and high-income classes corresponding to less than 400 Jordanian Dinar (JD), 400–800 JD, and more than 800 JD, respectively. Place of residence was classified into those living in the capital city,major cities other than the capital and countryside. Data about patients' diabetes-related care practices were collected at the same visit in which the questionnaire was collected, which included the frequency of self-monitoring of blood glucose (SMBG) and dietary compliance by counting carbohydrates or determining portions. Frequency was divided into always, mostly, rarely, and never, corresponding to daily, 4 to 6 days per week, less than 4 days per week, and none, respectively. Missing data, during review of the electronic charts, were collected during the clinic visit from patients and their parents.

Glycemic control was reflected by HbA1c at the time of clinic visit in which questionnaire was collected. HbA1c was expressed both as percentage (National Glycohemoglobin Standardization Program (NGSP)) and in mmol/mol (International Federation of Clinical Chemistry (IFCC)). It was measured by the Bio-Rad D-10™ Dual Program using ion-exchange high-performance liquid chromatography; normal reference range is 4.2-6.2% (22.41-44.27 mmol/mol).

### 2.1. Statistical Analysis

Statistical analysis was performed using IBM SPSS Statistics for Windows, version 23 (IBM Corp., Armonk, N.Y., USA). Continuous variables were presented as mean ± SD, and categorical variables were presented as frequencies (%). Associations between categorical variables were analyzed using the chi-squared test (*χ*^2^) or Fisher's exact test. Differences between groups of continuous variables were analyzed using independent samples *t* test. Possible predictors of depression were assessed using binary logistic regression. Variables including gender, duration of diabetes, HbA1c, hospital admission rate, frequency of daily glucose testing, compliance to counting carbohydrates, parental marital status, father's education level, mother's education level, and family monthly income were analyzed using univariate logistic regression, and possible predictors that were found to have *p* value < 0.25 were assessed by multivariable logistic regression analysis [24]. A *p* value < 0.05 was considered statistically significant. *P* values are not adjusted for multiple tests and therefore should be interpreted as exploratory only.

## 3. Results

A total of 108 children were enrolled in the study with an average age of 13.7 ± 2.3 years. Fifty-eight children (53.7%) had a CES-DC depression score less than 15, and 50 children (46.3%) had a depression score of 15 or more; mean scores were 7.9 ± 4.1 and 22.8 ± 7.1, respectively. Out of those with depression score of 15 or more, 41 children (38%) had a depression score between 15 and less than 30, 9 children (8.3%) had a depression score between 30 and less than 45, and none of the children had a score within the highest quartile (between 45 and 60).

The general characteristics of the two groups classified according to the CES-DC depression score are shown in Table 1.

The patients had an average duration of diabetes of 5.4 ± 3.2 years, and the mean BMI SDS was 0.21 ± 1.13. During the preceding year to the assessment of depressive symptoms, the average number of clinic visits was 4.8 ± 1.6. The average HbA1c for patients was 8.8 ± 1.3% (73.1 ± 14.8 mmol/mol). Differences in clinical characteristics among the 2 groups are shown in Table 2. There was no statistically significant difference in the mean age of the two groups, 13.6 ± 2.5 years for children with depression score less than 15 and 13.7 ± 2.1 years for those with depression score of 15 or more (*p* value = 0.894). Among the categorical variables, the number of diabetes-related hospital admissions divided by duration of diabetes and the number of daily glucose tests were significantly different between the two groups (Table 2).

To evaluate possible predictors of higher incidence of depressive symptoms, univariate and multivariable logistic regression analyses were performed. In the multivariable analysis, both gender and frequency of daily blood glucose tests were statistically significant with *p* values of 0.025 and 0.002, respectively. Girls were almost 3 and half times more likely to have a depression score ≥ 15 (OR = 3.41) than boys. Patients who were rarely testing blood glucose levels were more likely to have a depression score ≥ 15 compared to those who were testing regularly (OR = 36.57) (Table 3).

## 4. Discussion

Prevalence of significant depressive symptoms in patients with T1D in our study was 46.3%, which was relatively high, similar to other studies from developing countries, as those from India and Egypt, where prevalence rates were 36.9% [20] and 52.3% [21], respectively. Another study from Brazil showed a prevalence of depression of 53.6% in children and adolescents with T1D [25]. These studies reported a higher prevalence of depressive disorders than those reported in several studies from developed countries [13, 19, 26]. A study by K. Kokkonen and E. Kokkonen showed the presence of depression in 18% of adolescents with diabetes [27], which was at least double that of those without diabetes. Further, less prevalence of 12.9% and 12.3% in patients with T1D was reported by Adal et al. [28] and Jaser et al. [29], respectively.

Variable prevalence rates among different studies may be due to several factors including different screening tools used, different age groups of the study population, and the aim of a number of studies to investigate the prevalence of depression rather than depressive symptoms. In addition, several studies used wider age groups including patients with ages less than 10 years, such as the study from India [30], which interestingly showed a low prevalence of depression (7%) in their cohort compared to several studies from developed countries. This possibly was attributed to the fact that they had included a wider age range for patients from 4 to 15 years, compared to studies including adolescents who are known to have a higher prevalence of psychosocial disorders including depression. Furthermore, the authors explained that the low prevalence might be due to lower stress related to a less demanding insulin regimen of two injections of premixed insulin with less frequency of SMBG. In general, the relatively higher prevalence of depressive disorders in children and adolescents with T1D in developing countries is explained partly by the additional stress caused by limited resources and inadequate health care facilities for those patients [14]. Patients in our study faced additional challenges of living in a developing country with limited resources, especially that certain vital aspect in basic diabetes management; namely, devices needed for self-monitoring of blood glucose are covered partially for most patients and not covered at all for the rest of patients.

In our study, significant depressive symptoms were more prevalent among patients with a longer duration of T1D. Our findings were in line with another study that reported increased frequency of depressive disorders with longer duration of T1D with cumulative probability of major depressive disorder of 27.5% after 10 years from diagnosis [12]. On the other hand, there were studies that showed no correlation between the duration of diabetes and depressive symptoms [31, 32].

Patients with higher depressive scores in our study had less frequent self-blood glucose monitoring, higher HbA1c, and more diabetes-related hospital admissions, similar to other studies which found an association between low frequency of SMBG and higher incidence of depressive symptoms, with a resultant higher HbA1c [19, 33]. The diabetes-related hospital admissions were higher in patients with higher depression score, similar to what had been previously reported [21, 30]. It was found that the frequent number of hospital admissions was a predictor of psychological disorders [30]. Another study suggested that adolescents with multiple admissions for DKA should be screened for comorbid psychological disorders [34].

There was no significant difference in compliance with counting carbohydrate in our study between patients with lower and higher depression scores. On the other hand, other studies had shown a significant association between dietary noncompliance and a higher frequency of depressive symptoms [35]. Our results may be explained by the fact that dietary compliance was assessed as recalled by patients and their parents, which may be susceptible to bias.

Most of our patients were on multiple dose injection (MDI) therapy, and hence, there was no significant difference between the 2 groups classified according to depression score. In contrast, another study showed that patients on MDI had lower depression scores [21], which might be due, as they had explained, to the fact that those patients had better glycemic control with subsequent less stress.

We did not find a significant difference in glycemic control between pubertal and nonpubertal patients. It had been reported previously that depressive symptoms were higher in pubertal adolescents than those who did not reach puberty [21].

There were no significant differences in various socioeconomic elements between the 2 groups classified according to depression score, including monthly family income, place of residence, parental education level, and number of siblings. Percentage of married parents in our cohort was 95%, and all patients were covered by insurance. On the other hand, previous studies showed a correlation between socioeconomic factors and prevalence of depressive symptoms. A prospective study showed that lower socioeconomic status and living with single parent were significant factors for psychological issues in patients with T1D [36]. Those patients living with single parents had less financial resources and support needed for proper management of diabetes [37]. Also, it was found that parental involvement in diabetes care was a significant factor in commitment to the management plan put forward by the health care team [38]. It was noticed in some studies that higher parental education level was associated with lower prevalence of psychological disorders [20].

Predictors of a higher depression score in our study were female gender and less frequent SMBG. Duration of diabetes, HbA1c, and number of diabetes-related hospital admissions were significant predictors in univariate but not in multivariable regression analysis, which might be due to the confounding effect of other factors. Previous studies found a significant correlation between female gender and depressive disorders [13, 21]. Cohen et al. [36] reported that females were more susceptible to psychological issues, while other studies did not find such a correlation [20, 28, 39].

Higher HbA1c, in our study, was associated with higher depression scores. It was a significant predictor of depression on univariate but not on multivariable regression analysis, as previously mentioned. Our results were similar to those of a study which showed weak association between glycemic control and more prevalent depressive symptoms, but it was not a significant predictor of depression in the logistic regression analysis [28]. A meta-analysis revealed studies showing a significant association between glycemic control and psychological disorders, while others did not [40]. Khater and Omar reported a significant association between suboptimal glycemic control and a higher depression score; they showed that HbA1c was a significant predictor for depression among patients with diabetes using multivariable logistic regression [21]. The association between metabolic control as reflected by HbA1c and depression may be bidirectional. Studies done in adolescents with T1D investigating the association between glycemic control and depressive disorders were contradicting. Several studies showed significant associations [41], while no such correlation was evident in others [19, 37, 42, 43].

Failure to identify depressive symptoms in patients with T1D would have a significant effect on compliance to the required diabetes care plan and resultant suboptimal glycemic control [44, 45]. It is extremely important to identify patients with T1D at risk for depression as early as possible through regular evaluation for depressive symptoms during routine diabetes clinic visits [46].

The unfortunate lack in implementation of screening for depressive symptoms in the routine clinical diabetes care for patients with T1D may be due to limited resources, lack of prospective long-term studies confirming the significant association with glycemic control, reluctance of patients and/or their parents to respond to screening, and/or limited time available during the clinic visit. The significant high prevalence of depressive symptoms in patients with T1D as shown in our study and its association with suboptimal glycemic control highlights the importance of screening for depressive symptoms as early as possible. International guidelines recommend annual screening for depression in individuals with T1D using age-appropriate screening measures, taking into consideration that those with positive screening results need further evaluation [47].

This is the first study investigating depression in adolescents with T1D in Jordan. It adds to studies reported from other developing countries, where prevalence seems to be higher than that in developed countries and screening for depressive symptoms being unfortunately overlooked in the routine clinic follow-up visits. Efforts should be directed towards early screening of depressive symptoms in those patients shortly after diagnosis with T1D and then on regular intervals to look for the trend of the prevalence of depressive symptoms over time and for subsequent early referral to the proper psychiatric service for detailed evaluation and management.

We used CES-CD questionnaire in our study, while there were studies which used different tools as CES-D for adolescents with higher cutoff scores, which might had led to decreased reported frequency rates of depressive symptoms [48]. A number of contemporary research studies on depression screening in T1D are using the Patient Health Questionnaire-9 (PHQ-9) for adolescents, which is a shorter questionnaire with comparable specificity and sensitivity [49].

The results of our study had encouraged us to improve our care delivery practice, as we are planning to start screening for depressive symptoms in our patients with T1D who are older than 10 years, as part of routine care. Patients with higher depression scores would be given additional support by the pediatric diabetes care team, and parents should be advised to follow-up with pediatric mental health specialist for further evaluation.

There were a number of limitations of our study, which included the small sample size and the fact that the questionnaire was dependent on self-report of depressive symptoms, and the compliance to diabetes-related care practices was recalled by patients and their parents, which may be a cause of bias. Our screening was done in a single center in the capital city, which might not be representative of adolescents with T1D in other regions in the country. Future larger-scale longitudinal studies are needed, involving patients in variable cities to make more generalizable conclusions, rather than cross-sectional studies. In addition, a model using depression score as continuous ungrouped values would increase power and describes the association in a more unbiased method than using a binary cutoff point.

## 5. Conclusion

The prevalence of depression in adolescents with T1D in the pediatric population is relatively high, especially in developing countries. There was a significant association between depression and suboptimal glycemic control. The prevalence of significant depressive symptoms in patients with T1D was higher in those with longer duration of diabetes, higher HbA1c, and less frequent self-monitoring of blood glucose. Female gender was a significant predictor of depression in patients with TID. Regular screening of children and adolescents with diabetes for depressive symptoms should be part of their usual follow-up clinic visits. Patients with higher risk of developing depression should be identified for both their psychological health and diabetes management.

## Figures and Tables

**Table 1:** Differences in the demographic and socioeconomic characteristics between the 2 groups of patients in the study.

	Depression score < 15 (*N* = 58)	Depression score ≥ 15 (*N* = 50)	*p* value^∗^
Gender			0.069
Male	31 (53.4%)	18 (36.0%)	
Female	27 (46.6%)	32 (64.0%)	
Father's age			0.409
<50 years	37 (63.8%)	28 (56.0%)	
≥50 years	21 (36.2%)	22 (44.0%)	
Dead	0 (0%)	0 (0%)	
Father's education level			0.320
Less than high school	8 (13.8%)	4 (8.0%)	
High school	17 (29.3%)	21 (42.0%)	
Higher than high school	33 (56.9%)	25 (50.0%)	
Mother's age			0.232
<45 years	40 (69.0%)	27 (54.0%)	
≥45 years	17 (29.3%)	22 (44.0%)	
Dead	1 (1.7%)	1 (2.0%)	
Mother's education level			0.581
Less than high school	6 (10.3%)	10 (20.0%)	
High school	24 (41.4%)	18 (36.0%)	
Higher than high school	27 (46.6%)	21 (42.0%)	
Dead	1 (1.7%)	1 (2.0%)	
Place of residence			0.341
Capital Amman	44 (75.9%)	35 (70.0%)	
Major city other than the capital	7 (12.1%)	4 (8.0%)	
Countryside	7 (12.1%)	11 (22.0%)	
Family monthly income (Jordanian dinars)			0.734
<400	11 (19.0%)	11 (22.0%)	
400–800	40 (69.0%)	31 (62.0%)	
>800	7 (12.1%)	8 (16.0%)	
Number of siblings			0.542
≤4	49 (84.5%)	40 (80.0%)	
>4	9 (15.5%)	10 (20.0%)	

^∗^Chi-squared test (*χ*^2^).

**Table 2:** Differences in the clinical and diabetes care practice characteristics between the 2 groups of patients in the study.

	Depression score < 15 (*N* = 58)	Depression score ≥ 15 (*N* = 50)	*p* value^∗^
*Categorical variables*			
Pubertal status			0.203
Yes	35 (60.3%)	36 (72.0%)	
No	23 (39.7%)	14 (28.0%)	
Type of insulin regimen			0.805
Multiple dose injection	53 (91.4%)	45 (90.0%)	
Triple dose injection	5 (8.6%)	5 (10.0%)	
Compliance to counting carbohydrate			0.321
Always/mostly	39 (67.2%)	29 (58.0%)	
Never/rarely	19 (32.8%)	21 (42.0%)	
Who decide insulin dose			0.938
Mother	41 (70.7%)	35 (70.0%)	
Patient herself/himself	17 (29.3%)	15 (30.0%)	
Hospital admission rate			0.013
0 (no admission)	47 (81.0%)	30 (60.0%)	
<1	10 (17.2%)	20 (40.0%)	
>1	1 (1.7%)	0 (0%)	
Frequency of daily glucose testing			0.001
Always/mostly	57 (98.3%)	32 (64.0%)	
Rarely/never	1 (1.7%)	18 (36.0%)	

	Depression score < 15 (*N* = 58)	Depression score ≥ 15 (*N* = 50)	*p* value^∗∗^
*Continuous variables*			
HbA1c (%) (mmol/mol)	8.6 ± 1.3 (70.4 ± 13.8)	9.1 ± 1.3 (76.2 ± 14.3)	0.034
Current BMI *z*-score	0.09 ± 1.17	0.35 ± 1.07	0.249
Duration of diabetes (years)	4.9 ± 2.7	6.1 ± 3.5	0.041
Number of clinic visits over the last year	4.7 ± 1.4	5.0 ± 1.8	0.355

^∗^Chi-squared test (*χ*^2^). ^∗∗^Independent samples *t* test.

**Table 3:** Univariate and multivariable logistic regression analysis for possible predictors of depression.

	Univariate logistic regression			Multivariable logistic regression		
	Odds ratio	95% CI	*p* value	Odds ratio	95% CI	*p* value
Gender						
Male (reference)						
Female	2.04	0.94-4.43	0.071	3.41	1.16-9.99	0.025
Duration of diabetes	1.14	1.01-1.30	0.041	1.09	0.91-1.29	0.349
HbA1c (%)	1.39	1.02-1.90	0.038	1.31	0.87-1.95	0.193
Hospital admission rate						
0 (reference)						
<1	3.13	1.29-7.60	0.012	1.73	0.56-5.32	0.338
Frequency of daily glucose testing						
Always/mostly (reference)						
Rarely/never	32.06	4.09-251.48	0.001	36.57	3.79-352.79	0.002
Compliance to carbohydrates count						
Always/mostly (reference)						
Never/rarely	1.45	0.66-3.18	0.356			
Parental marital status						
Married (reference)						
Divorced or separated	1.15	0.16-8.48	0.892			
Dead parent	1.15	0.07-18.89	0.923			
Father's education level						
Less than high school (reference)						
High school	2.47	0.63-9.62	0.192	3.70	0.50-27.12	0.198
Higher than high school	1.52	0.41-5.60	0.534	3.13	0.38-25.73	0.289
Mother's education level						
Less than high school (reference)						
High school	0.45	0.14-1.47	0.186	0.28	0.05-1.52	0.141
Higher than high school	0.47	0.15-1.49	0.199	0.24	0.04-1.52	0.131
Dead	0.60	0.03-11.47	0.734	0.20	0.004-9.027	0.407
Family monthly income (Jordanian dinars)						
<400 (reference)						
400–800	0.78	0.20-2.02	0.602			
>800	1.33	0.35-5.14	0.676			

## Data Availability

The data that support the findings of this study are available on request from the corresponding author. The data are not publicly available due to privacy or ethical restrictions.
